# Light in the Sick Room

**Published:** 1885-05

**Authors:** 


### Light in the Sick-Room.

It is the unqualified result of all my experience with the sick, that second only to their need of fresh air is their need of light; that, after a close room, what hurts them most is a dark room, and that it is not only light but direct sunlight they want. You had better carry your patient about after sun, according to the aspect of rooms, if circumstances permit, than let him linger in a room when the sun is off. People think that the effect is upon the spirits only. This is by no means the case. Who has not observed the purifying effects of light, and especially of direct sunlight, upon the air of a room? Here is an observation within everybody's experience. Go into a room where the shutters are always shut (in a sick-room there should never be shutters shut), and though the room be uninhabited, though the air has never been polluted by the breathing of human beings, you will observe a close, musty smell of corrupt air—of air unpurified by the effect of the sun's rays. The mustiness of dark rooms and corners, indeed, is proverbial. The cheerfulness of a room, the usefulness of light in treating disease, is all-important. It is a curious thing to observe how almost all patients lie with their faces turned to the light, exactly as plants always make their way towards the light.—*Florence Nightingale's Notes of Nursing.*

